# Can Na^**18**^F PET/CT Be Used to Study Bone Remodeling in the Tibia When Patients Are Being Treated with a Taylor Spatial Frame?

**DOI:** 10.1155/2014/249326

**Published:** 2014-03-19

**Authors:** Henrik Lundblad, Gerald Q. Maguire, Henrik Olivecrona, Cathrine Jonsson, Hans Jacobsson, Marilyn E. Noz, Michael P. Zeleznik, Lars Weidenhielm, Anders Sundin

**Affiliations:** ^1^Department of Molecular Medicine and Surgery, Section of Orthopaedics and Sports Medicine, Karolinska Institute, A2:07, 171 76 Stockholm, Sweden; ^2^School of Information and Communication Technology, KTH Royal Institute of Technology, Isafjordsgatan 26, 418 164-40 Stockholm, Sweden; ^3^Department of Hospital Physics, Karolinska University Hospital, 17 176 Solna, Stockholm, Sweden; ^4^Department of Radiology, New York University, 550 First Avenue, THW232, New York, NY 10016, USA; ^5^School of Computing, College of Engineering, University of Utah, 50 Central Campus Dr., Room 3190, Salt Lake City, UT 84112, USA

## Abstract

Monitoring and quantifying bone remodeling are of interest, for example, in correction osteotomies, delayed fracture healing pseudarthrosis, bone lengthening, and other instances. Seven patients who had operations to attach an Ilizarov-derived Taylor Spatial Frame to the tibia gave informed consent. Each patient was examined by Na^18^F PET/CT twice, at approximately six weeks and three months after the operation. A validated software tool was used for the following processing steps. The first and second CT volumes were aligned in 3D and the respective PET volumes were aligned accordingly. In the first PET volume spherical volumes of interest (VOIs) were delineated for the crural fracture and normal bone and transferred to the second PET volume for SUV_max_ evaluation. This method potentially provides clinical insight into questions such as, when has the bone remodeling progressed well enough to safely remove the TSF? and when is intervention required, in a timelier manner than current methods? For example, in two patients who completed treatment, the SUV_max_ between the first and second PET/CT examination decreased by 42% and 13%, respectively. Further studies in a larger patient population are needed to verify these preliminary results by correlating regional Na^18^F PET measurements to clinical and radiological findings.

## 1. Introduction

Circular frames, such as the Ilizarov-derived Taylor Spatial Frame (TSF), with the ability to correct deformity in six dimensions have added new possibilities to treat difficult orthopaedic conditions [1–3]. Complex open fractures, malunions, infected nonunions, and severe deformities are all examples of conditions where the circular frame has enabled limb salvage as opposed to amputation [4–6]. Despite the obvious advantages of being able to gradually correct deformity, without having to go back to the operating theater, circular frames are currently mainly reserved for more complex conditions. In these cases, it is common that patients undergo TSF treatment for more than 12 months. Apart from the inconvenience of bearing a bulky external fixator this long, the treatment may be painful; there is a risk of opioid addiction and possible psychosocial and occupational problems for the patient.

Therapy using a TSF is performed according to a treatment schedule, which is a set of daily instructions for mechanical adjustments to the struts of the frame. These mechanical adjustments bring the bone fragments into their final desired alignment in six dimensions (i.e., both translations and rotations) [7]. Computed tomography (CT) together with planar X-ray imaging and clinical examinations are currently used for preoperative evaluation and to detect the need for revision surgery during the course of treatment. As bone remodeling is dependent on stable fixation and adequate blood supply to viable bone, the initial surgery is of critical importance to minimize the risk of reoperations with bone grafting or stabilization of the frame. Current radiological techniques are unable to predict the healing potential at an early stage. If patients with a high risk of delayed or nonunion could be identified early during the treatment or even preoperatively, late revisions leading to prolonged treatments and unnecessary late amputations might be avoided.

For the last ten years CT together with 3D volume rendering techniques (VRT) has been applied to total hip arthroplasty (THA) [8–11]. We previously evaluated TSF treatment progression using CT [12]. Bone remodeling relative to THA has been investigated using sodium ^18^flouride (Na^18^F) positron emission tomography (PET) [13]. The improvements in CT and PET imaging, the earlier use of Na^18^F in bone scanning, the ready availability of cyclotron produced Na^18^F, our experience in multimodality image processing, and the needs of the orthopedic surgeons to deal with complex tibia cases suggested that evaluating TSF treatment progression using Na^18^F PET could be insightful. However, the effect of metal artifacts in the CT examination on the PET attenuation correction needed to be assessed and a suitable reconstruction algorithm needed to be determined; hence initial phantom studies were performed [14]. Issues regarding metal artifacts have also been investigated by others [15, 16]. Based upon the results of these phantom studies and our initial work [14] a suitable imaging protocol was designed. In this paper we describe how Na^18^F PET/CT can help in evaluating TSF treatment progression in a number of very different orthopaedic conditions.

## 2. Methods and Materials

### 2.1. Patients

Seven of eight consecutive patients, 5 males and 2 females (mean age 36, range 17–52 years), who had operations (between September 2012 and June 2013) to attach a TSF to the tibia gave informed consent to participate in this study which was approved by the Regional Ethics Committee (Dnr 2012/1049-31/1). There were no other selection criteria. One patient was having both legs treated for Genu Varum; thus eight legs were studied in total. Two Na^18^F PET/CT examinations were performed on each patient, first at approximately six weeks (range 40–60; mean 49 days) and again approximately three months (range 84–184; mean 113 days) after the operation. The time points of six weeks and three months were chosen because the major bone remodeling was expected to occur early. With the exception of Patient four, who was operated on again 77 days after the first operation (25 days after the first Na^18^F PET/CT examination and 17 days before the second), none of the other patients were operated on between examinations. Additionally, two male patients aged 64 and 36 years were clinically examined to determine their bone remodeling at a time close to the removal of the TSF. These two Na^18^F PET/CT examinations were performed at 274 and 135 days after the initial operation and the frame was removed at 328 and 211 days, respectively.  Table 1 gives a detailed description of all the patients.

### 2.2. Na^18^F PET/CT Examinations

A clinical PET/CT scanner (Biograph 64 TruePoint TrueV, Siemens Medical Solutions, Erlangen, Germany) was used for all examinations. The patient was positioned supine on the scanning couch as described in [14] to include the tibia at the location of the crural fracture which included some or all of the TSF in the axial field of view so that one bed position (20 cm) could be used for the PET (i.e., the scanning couch need not be moved). An anterioposterior scout view (CT topogram) was performed as described in [14]. A dynamic PET acquisition was performed in list mode, was started simultaneously with the intravenous Na^18^F injection (2 MBq/kg body weight) and continued for 45 minutes, and was followed by a five-minute static scan started at 60 minutes after tracer injection. The list mode acquisition procedure was performed to determine the best time frame to provide data for a suitable reconstruction. This dynamic acquisition also provided data for the imaging of tracer transport from blood to the fracture site. This, however, has not yet been done [17]. The PET data were reconstructed using the parameters shown in Table 2. Two volumes at 30 and at 45 minutes after injection were reconstructed from the list mode data as suggested by [17], and a third volume was reconstructed from the five-minute static scan acquired after 60 minutes. As the patient was not moved during the entire acquisition time, a single CT scan was used for attenuation correction of the two acquisitions. However, two patients had moved slightly between the list mode and static examinations, so a second CT scan was performed with the same exact parameters of the first scan (see Table 2).

### 2.3. Image Analysis

A 3D image processing software tool, described and validated elsewhere [8, 18, 19], was used as follows for each patient. The CT and PET volume data from the first and second PET/CT examinations were spatially aligned into a single coordinate system. This was done by manually selecting physiologically guided landmarks on each tibia close to the crural fracture and on the pins in both the first and second CT diagnostic volumes (277 slices). From these landmarks a registration algorithm created a rigid body transformation which was used to bring the second CT volume into alignment with the first one (for both the diagnostic and attenuation correction reconstructions). Using numerous evaluation tools (2D and 3D, visual and quantitative), the landmarks and transformation were tuned until acceptable. This same transformation was then applied to bring the second PET volume into alignment with the first one. As the results of this alignment were not perfect (because the CT-CT alignment and the original CT-PET alignment from the examination were not perfect) the final PET volume alignment was refined and evaluated with manual adjustments using the software. Once all volumes were considered to be in the same coordinate system, then the subsequent volumes of interest (VOIs) and standardized uptake values (SUV) are derived from the same physiological volume.

Spherical VOIs were created with a 3D spherical landmark tool. Using the first PET volume, the crural fracture corresponding to the remodeling volume on the CT and with the highest tracer uptake was outlined with a spherical VOI of 40 mm diameter. This VOI was then transferred to the second PET volume. Both PET volumes and the corresponding CT diagnostic volumes were then superimposed, and the evaluation tools were used to confirm the correct placement of the VOI in both PET/CT volumes. VOIs with 20 mm diameter were placed on the PET volume in the contralateral tibia to include what was presumed to be normal bone. In the one patient who had both tibiae treated, the normal bone VOI was placed on a portion of each tibia as far as possible from the crural fracture and the pins. The maximum, minimum, mean, and median SUV for each VOI were recorded and saved in a comma separated values (CSV) file. The complete data from the SUV calculation on each voxel in the VOIs was also saved in a separate CSV file.

The magnitude of these results was contrasted with that obtained by using a similar tool provided by the Siemens syngo MultiModality Workplace (syngo MMWP VE36A), to check that our tool was operating properly. However, the presence of the attached TSF made it difficult for the commercial software provided in the Siemens workstation to satisfactorily align the tibia between the volumes obtained at two different times.

## 3. Results

**Figure 1:**
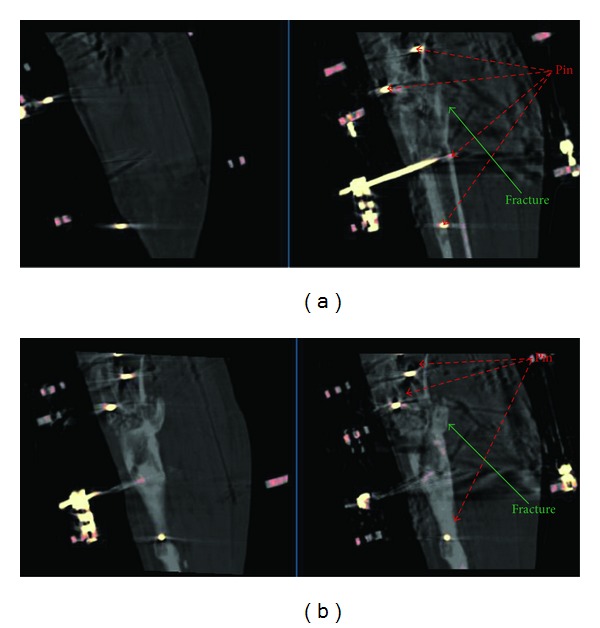
A sagittal CT section for Patient 1 showing the original CT misalignment between the first CT at 40 days (right) and the second CT at 84 days (left) after the operation to attach the TSF (a). A sagittal CT section for Patient 1 showing the final CT transformed alignment between the first CT at 40 days (right) and the second CT at 84 days (left) after the operation to attach the TSF (b).

**Figure 2:**
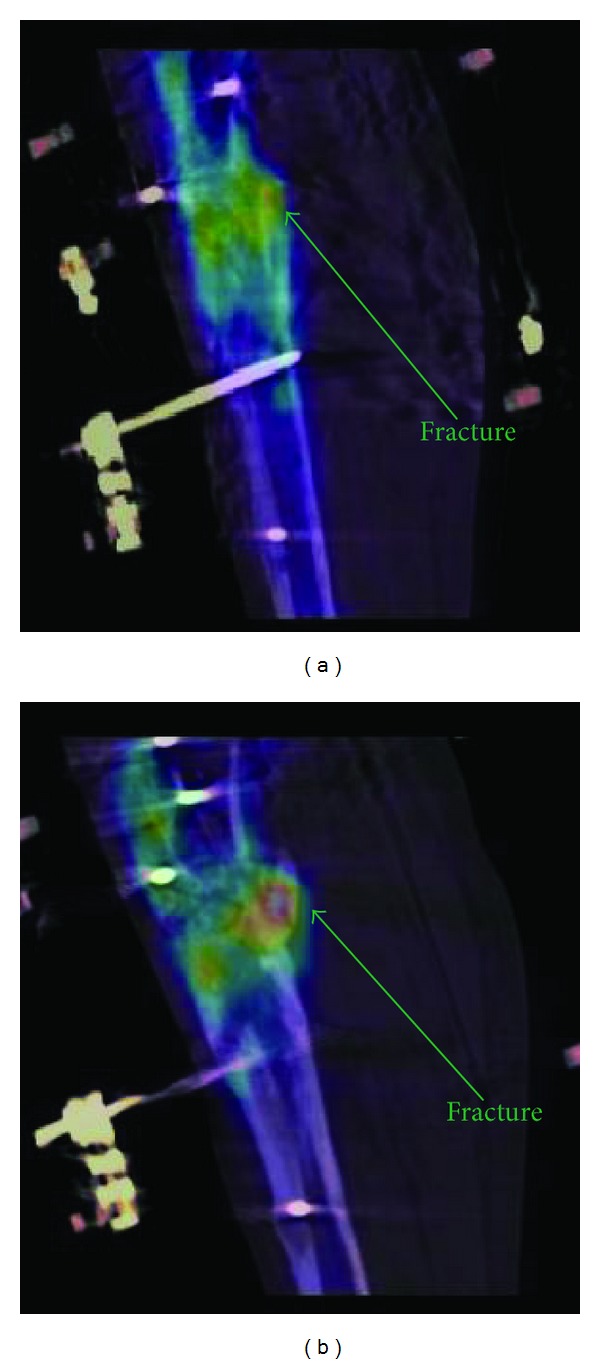
A sagittal fused PET/CT section of the crural remodeling area for Patient 1 at 40 days (a) and at 84 days (b) after the initial operation to attach the TSF. The crural remodeling region on the PET is better aligned to the CT in (a) than in (b).

**Figure 3:**
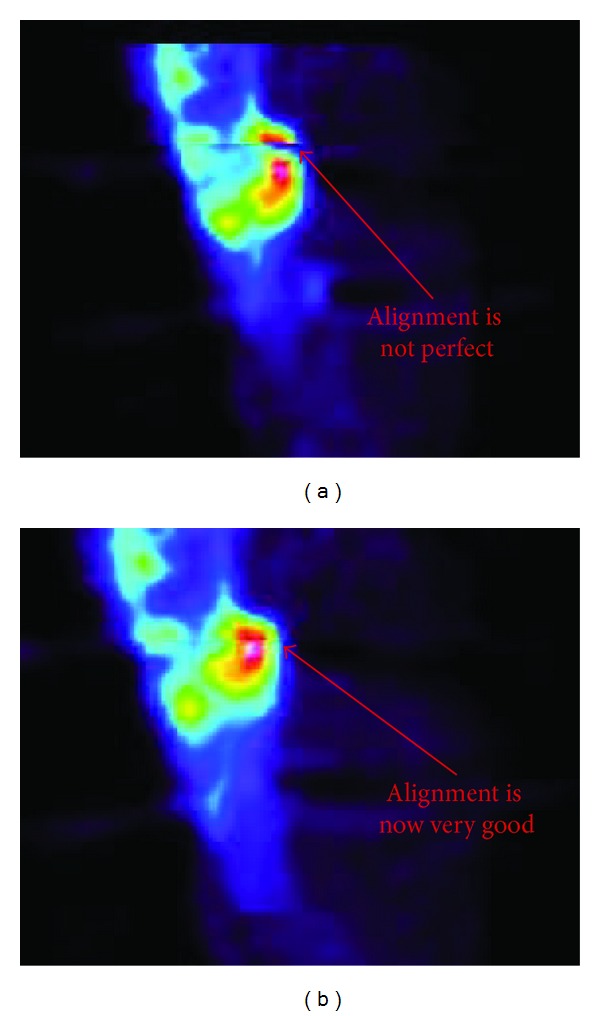
A sagittal section of the first PET scan for Patient 1 is superimposed on the second one. The first alignment between the exam at 40 days and at 84 days is shown in (a) and the final alignment after a slight manual adjustment in (b). This allows the same VOI to be used for the SUV calculations.

In all patients, the PET/CT volume alignment could be performed and was visually checked both by using coronal, sagittal, and axial projections (viewed side by side or superimposed) and by superimposing the two 3D volumes (viewed as 3D isosurfaces). Based on distance difference calculations performed on the transformed landmarks (transformed moving landmarks subtracted from reference landmarks), the mean landmark error for each individual CT-CT alignment was within one PET voxel (6.5 mm) and the mean landmark error for all CT-CT alignments was 2.4 mm. An example of the original misalignment between the first and second CT examinations is shown in Figure 1(a) with the final transformed alignment in Figure 1(b). The CT-PET alignment for the first examination is shown in Figure 2(a) and for the second examination in Figure 2(b). The PET and CT volumes were aligned, but not perfectly, especially as visible in the second examination (Figure 2(b)). As a result of this slight misalignment, the corresponding PET volumes were not perfectly aligned after applying the CT landmark transformation (Figure 3(a)). However, after applying manual adjustments, a suitable final alignment was obtained (Figure 3(b)).

**Figure 4:**
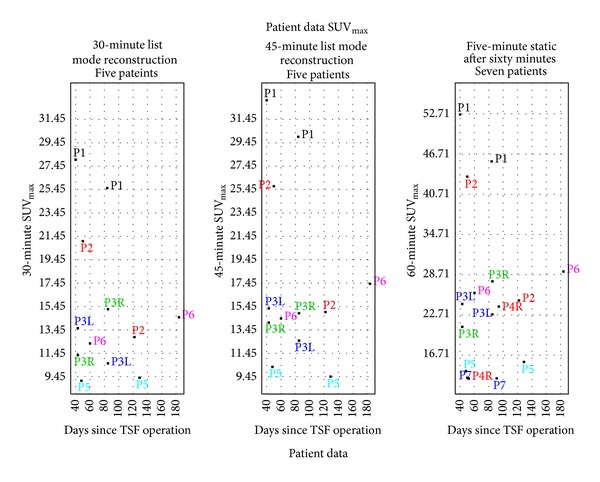
The SUV_max⁡_ for the leg which has the TSF applied plotted as a function of days after the first application of the TSF. Graphs are drawn for five study patients for the 30- and 45-minute reconstructions from list mode using the same scale. A graph is drawn for the seven study patients for the 5-minute static scan taken after 60 minutes on an expanded scale.

The SUV_max⁡_ for the crural fracture and reference bone region are shown in Table 3.  Figure 4 gives a graphical presentation of the SUV_max⁡_ for the 30-minute and 45-minute volume data reconstructed from the list, as well as the five-minute static examination acquired after 60 minutes. As the list mode data was not available from the archive for the first study of Patient 4 and the second study of patient 7, only five patients are included in the 30- and 45-minute graph.

**Figure 5:**
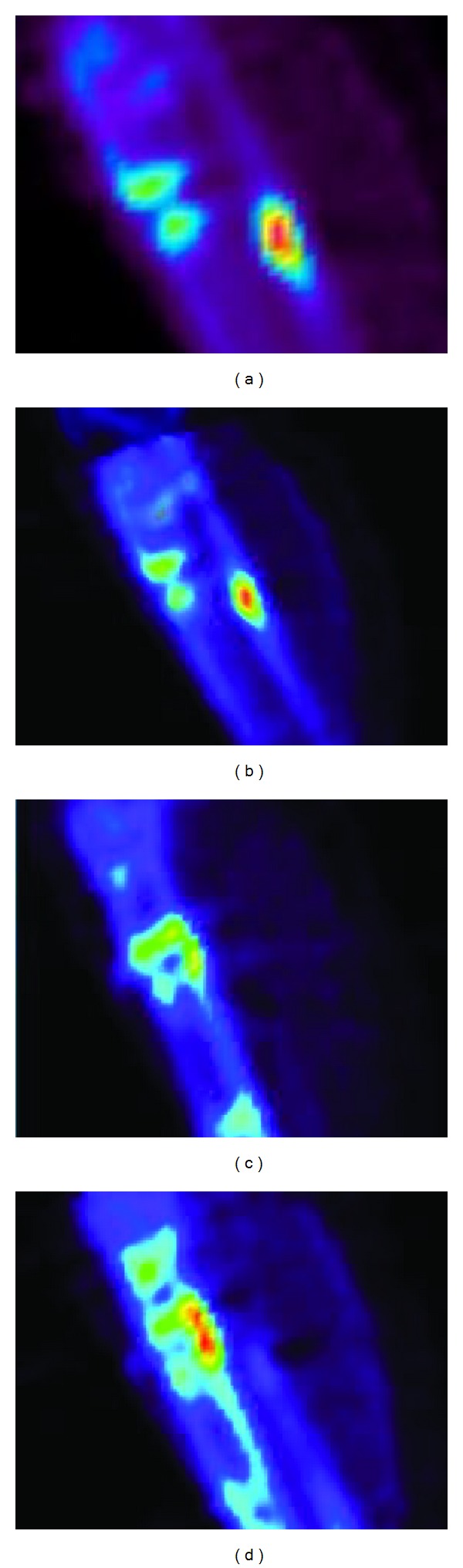
A sagittal section for Patient 4 of the PET at 52 days (a) and at 94 days (b) showing the fibula. A sagittal section of the PET at 52 days (c) and at 94 days (d) showing the tibia. The remodeling of the tibia in (d) seems to be uneven.

**Figure 6:**
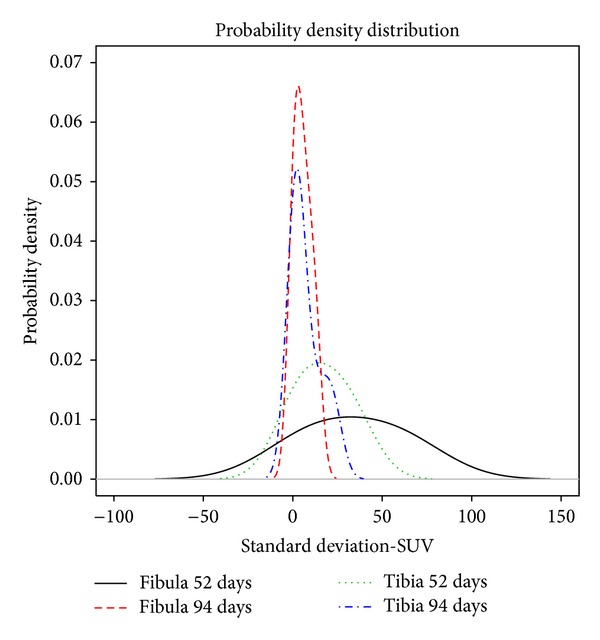
The probability density plot showing the density distribution of SUV data for Patient 4. The second tibia distribution shows that the data may consist of two density distributions as was suspected from the clinical findings.

The clinical results for the patients are summarized in Table 1. One example of an interesting result is that of Patient 4. This patient had a reduction malformation of the right leg. She was treated with a TSF and a percutaneous osteotomy of the tibia and an oblique osteotomy of the fibula. Fifty-two days postoperatively the tibia osteotomy SUV_max⁡_ was 13.7, while the fibula osteotomy SUV_max⁡_ was 66.0. Two months (56 days) postoperatively it was observed that her fibula had healed prematurely and she was therefore reosteotomized. Two weeks later (94 days after the first operation and 17 days after the second) the tibia osteotomy SUV_max⁡_ had increased to 24.5 and the fibula osteotomy SUV_max⁡_ had decreased to 36.5. This SUV data may be related to the functionality and underlying process occurring within the bone cells at 54 and 96 days. For the fibula, the visual presentation of the PET data (Figures 5(a) and 5(b)) and the probability density function of the standard deviation of the SUV data (Figure 6) were similar at 54 and 96 days. At 54 days there was a broad spectrum of intensity and at 94 days there was a narrow intense spectrum. For the tibia at 52 days the PET visually shows two moderately intense areas (Figure 5(c)), but when the density function of the standard deviation of the SUV data was graphed (Figure 6) there is a continuous normal probability density presented by the data; the populations are equal in intensity and overlap. In contrast, the PET presents a large visually intense region (Figure 5(d)) at 94 days. The probability density function of the standard deviation of the SUV data (Figure 6) presents two populations, one at higher intensity which overlaps part of the second.

## 4. Discussion

To our knowledge this is the first study showing that Na^18^F PET/CT is applicable to assess treatment progression in patients being treated with a TSF. There are several sources of difficulty in the PET/CT volume analysis. The metal in the TSF causes CT artifacts which make landmark selection challenging in the CT volumes. Additionally, the positions of the pins and the bones are shifted daily (with possibly six degrees of motion) over the course of the therapy by the mechanical strut adjustments that are performed by the patient during treatment. Despite the fact that both the bone and the various parts of the TSF shifted between the baseline and follow-up Na^18^F PET/CT, it was possible to align the VOIs in the examinations using our software within one-PET-voxel accuracy, so that differential PET measurements could be made. As can be seen from Figure 4, in all cases the SUV_max⁡_ is higher at 60 minutes than at 30 and 45 minutes. However, as both sensitivity and specificity for bone is high for Na^18^F PET and it has a rapid uptake as well as a high contrast bone-to-background it is possible to use the SUV_max⁡_ obtained at 30 minutes or at 45 minutes, if it is not possible to wait for the full 60 minutes. This potentially enables a Na^18^F PET/CT examination of TSF patients in the morning before the scanning of F-18 fluorodeoxyglucose (FDG) patients begins (assuming that the scanner and personnel are available).

The Na^18^F PET/CT examinations provided morphological as well as quantitative information regarding regional bone turnover [20, 21] that has a potential to substantially shorten ineffective treatments by earlier detection of a probable nonunion and may provide a means to monitor the distraction rate in bone lengthening. While the method described here provides a precise technique to assess Na^18^F uptake/bone turnover, we still know very little about how to use this information. Some possible uses include performing a preoperative Na^18^F PET/CT scan to aid in preoperative planning; the method might be used to determine the viability of the bone or to indicate the healing potential and the need for either additional stability or for an autologous bone graft.

From the preliminary data acquired for Patients 6 and 7, a low Na^18^F uptake shortly after surgery can indicate potentially poor healing and prompt early intervention. Also, we observed that in the first two patients who healed and had the frame removed during the course of the study, the SUV_max⁡_ decreased by 42 and 13%, respectively. This indicates that it is of interest to monitor uptake of Na^18^F by PET/CT before and during different adjuvant treatments to assess its potential as tool for evaluating the ability of TSF treatment to stimulate bone remodeling.

In leg lengthening it is important that the distraction rate is adjusted during treatment to prevent both nonunion and premature healing. The callus formation seen on a planar X-ray is quite late and sometimes difficult to evaluate. In contrast, Na^18^F PET/CT can provide quantitative information regarding the regional bone remodeling. It may be hypothesized that if the maximum Na^18^F uptake is separated into a distal and a proximal region (as opposed to one homogenous region) this may indicate that the lengthening is occurring too rapidly.

Our results lead to a number of clinical questions that need to be answered in future studies. For example, when has the bone remodeled sufficiently to safely remove the TSF? As a more long term goal we would like to understand if the information from the Na^18^F PET/CT examination could be used to find a patient specific bone remodeling model that could be used to optimize the TSF treatment from a* functional* point of view; in a fashion similar to the CT/planar X-ray* morphological* data is used today for TSF treatment planning, thus potentially reducing the duration of the TSF treatment.

## 5. Conclusions

This study has shown that Na^18^F PET/CT can be used to quantitatively assess how the TSF influences bone remodeling by providing information that is not revealed by conventional radiological examinations. Although limited to a small number of patients in this exploratory setting, information, which could have had an impact on patient management, was gained. The study also pointed out a number of clinical applications of this method that would be useful in evaluating an individual patient's progression.

## Figures and Tables

**Table 1:** Patient description (N/A means not applicable).

Patient	Age	Sex	Days first PET/CT	Days second PET/CT	Reason	Resolution	Days TSF applied
P1	52	M	40	84	Fracture of upper end of tibia, closed; delayed fracture healing in left leg	TSF extraction healed	167
P2	44	M	50	122	Pseudarthrosis right lower leg	TSF extraction healed	161
P3	35	M	43	85	Genu Varum (bow leg), pseudoachondroplasia	TSF extraction healed	182
P4	17	F	52	94	Reduction malformation right lower leg	TSF extraction healed	345
P5	31	M	48	129	Fracture of upper end of tibia, closed, osteomyelitis right lower leg	Patient chose to have leg amputated because of continued infection	226
P6	28	M	60	184	Fracture of upper end of tibia, closed, tendon trouble for other fractures of the lower extremity, infected pseudarthrosis left lower leg, remaining foreign body in the soft tissue	Patient was monitored with planar X-ray imaging and was fully weight bearing and painless after several weeks. However, a CT scan showed a hypertrophic nonunion. He is now planned for lengthening of the tibia proximally and compression/stabilization of the nonunion	N/A
P7	45	F	50	91	Nonunion/pseudarthrosis distal tibia/pilon fracture right mechanical complication of osteosynthesis (broken bolts) right distal tibia	Planar X-ray was *not* able to identify any healing disturbances; however, a CT clearly showed a nonunion. The low uptake at 50 days perhaps should have been an indication that an early revision with autologous bone graft would have been beneficial to the patient	N/A
P8	64	M	274	N/A	Refracture in segmental tibial fracture on the left leg	TSF extraction proximal tibia healed—applied cast to distal tibia	328
P9	36	M	135	N/A	Pseudarthrosis right lower leg	TSF extraction healed	211

**Table 2:** PET and CT reconstruction parameters.

	Modality		Resolution			Pixel size (mm)		
	Parameters	Reconstruction	*X*	*Y*	*Z*	*X*	*Y*	*Z*
PET	Dynamic list mode and static mode	OSEM2D Four iterations Eight subsets	168	168	74	4.07	4.07	3.00

CT	120 kV, 60 mA 0.5 second per revolution 1.0 pitch	Diagnostic Attenuation correction	512 512	512 512	277 74	0.98 1.37	0.98 1.37	0.80 3.00

**Table 3:** Maximum SUV at two different times. The patient (P3) with both legs treated is marked with P3 L (left) and P3 R (right) and the patient with very active bone turnover in the fibula is marked with P4 R (right) and P4 F (fibula).

Patient	Days TSF surgery	Operated leg SUV_max_	Nonoperated leg SUV_max_
P1	40	53.1	2.2
P1	84	46.1	1.2
P2	50	43.8	2.2
P2	122	25.4	3.5
P3 L	43	24.8	1.0
P3 L	85	23.3	2.7
P3 R	43	21.4	1.7
P3 R	85	28.2	1.8
P4 R	52	13.7	4.3
P4 R	94	24.5	1.7
P4 F	52	66.0	2.0
P4 F	94	36.5	2.0
P5	48	14.8	1.1
P5	129	16.2	2.0
P6	60	26.5	1.7
P6	184	29.7	1.8
P7	50	13.8	1.6
P7	91	13.8	1.5
